# Application and challenges of TCR and BCR sequencing to investigate T- and B-cell clonality in elastase-induced experimental murine abdominal aortic aneurysm

**DOI:** 10.3389/fcvm.2023.1221620

**Published:** 2023-11-14

**Authors:** Christin Elster, Miriam Ommer-Bläsius, Alexander Lang, Tanja Vajen, Susanne Pfeiler, Milena Feige, Tin Yau Pang, Marius Böttenberg, Sarah Verheyen, Khang Lê Quý, Maria Chernigovskaya, Malte Kelm, Holger Winkels, Susanne V. Schmidt, Victor Greiff, Norbert Gerdes

**Affiliations:** ^1^ Division of Cardiology, Pulmonology, and Vascular Medicine, Medical Faculty and University Hospital, Heinrich Heine University, Düsseldorf, Germany; ^2^ Department of Biology, Institute for Computer Science, Heinrich Heine University, Düsseldorf, Germany; ^3^ Department of Immunology, University of Oslo and Oslo University Hospital, Oslo, Norway; ^4^ Cardiovascular Research Institute Düsseldorf (CARID), Medical Faculty, Heinrich Heine University, Düsseldorf, Germany; ^5^ Department of Cardiology, Faculty of Medicine and University Hospital Cologne, University of Cologne, Cologne, Germany; ^6^ Institute of Innate Immunity, Medical Faculty and University Hospital, Rheinische Friedrich-Wilhelms-University, Bonn, Germany; ^7^ Institute of Clinical Chemistry and Clinical Pharmacology, University Hospital Bonn, Bonn, Germany

**Keywords:** aortic aneurysm, single-cell sequencing (scRNA-seq), T cell receptor (TCR), B cell receptor (BCR), clonality analysis

## Abstract

**Background:**

An abdominal aortic aneurysm (AAA) is a life-threatening cardiovascular disease. Although its pathogenesis is still poorly understood, recent evidence suggests that AAA displays autoimmune disease characteristics. Particularly, T cells responding to AAA-related antigens in the aortic wall may contribute to an initial immune response. Single-cell RNA (scRNA) T cell receptor (TCR) and B cell receptor (BCR) sequencing is a powerful tool for investigating clonality. However, difficulties such as limited numbers of isolated cells must be considered during implementation and data analysis, making biological interpretation challenging. Here, we perform a representative single-cell immune repertoire analysis in experimental murine AAA and show a reliable bioinformatic processing pipeline highlighting opportunities and limitations of this approach.

**Methods:**

We performed scRNA TCR and BCR sequencing of isolated lymphocytes from the infrarenal aorta of male C57BL/6J mice 3, 7, 14, and 28 days after AAA induction via elastase perfusion of the aorta. Sham-operated mice at days 3 and 28 and non-operated mice served as controls.

**Results:**

Comparison of complementarity-determining region (CDR3) length distribution of 179 B cells and 796 T cells revealed neither differences between AAA and control nor between the disease stages. We found no clonal expansion of B cells in AAA. For T cells, we identified several clones in 11 of 16 AAA samples and one of eight control samples. Immune receptor repertoire comparison indicated that only a few clones were shared between the individual AAA samples. The most frequently used V-genes in the TCR beta chain in AAA were TRBV3, TRBV19, and the splicing variant TRBV12-2 + TRBV13-2.

**Conclusion:**

We found no clonal expansion of B cells but evidence for clonal expansion of T cells in elastase-induced AAA in mice. Our findings imply that a more precise characterization of TCR and BCR distribution requires a more extensive number of lymphocytes to prevent undersampling and potentially detect rare clones. Thus, further experiments are necessary to confirm our findings. In summary, this paper examines TCR and BCR sequencing results, identifies limitations and pitfalls, and offers guidance for future studies.

## Introduction

An abdominal aortic aneurysm (AAA) is a cardiovascular disease characterized by a permanent dilation of the abdominal aorta greater than 50% or 3 cm. Most AAAs develop in the infrarenal region between the renal veins and the aortic bifurcation (1). The prevalence of AAA is 4%–8% in men older than 60 years and 0.5%–1.5% in women, with the rupture of AAA conferring a high mortality rate (2). AAA is a multifactorial and progressive disease. Genetic factors and inflammation strongly contribute to AAA development (3), and several studies have revealed recently that autoimmunity may contribute to the pathogenesis of AAA (4–8).

Inflammation and immune cell recruitment are characteristics of AAA. Accordingly, T and B cells are among the predominant infiltrating immune cells in human AAA tissue (4, 8, 9). The presence of these lymphocytes in AAA tissue was confirmed in several experimental mouse models of AAA including the porcine pancreatic elastase (PPE) perfusion model, which was used for this study (10–14). The PPE model produces infrarenal aortic aneurysms and is considered the experimental mouse model most resembling human AAA, although the aneurysms do not form intraluminal thrombus or rupture (15). Several experimental interventions (e.g., HIF-1α inhibitors, PI3Kγ inhibitors) preventing elastase-induced AAA are associated with decreased numbers of lymphocytes in the aneurysmal tissue (16–18) (Supplementary Table S1), suggesting an important role for lymphocytes in AAA development in this model.

Previous reports have implicated both T helper-1 (Th1) and T helper-2 (Th2) cells in various stages of AAA development (19). Th2 cells release inflammatory mediators and cytokines such as interleukins 4, 5, 9, 10, and 13 and Fas ligand that may contribute to the regulation of AAA progression, whereas Th1-derived interferon-gamma and CD40 ligand are associated with macrophage activation, regulation of vascular smooth muscle cell apoptosis, and aortic wall remodeling (2, 19–22). Investigation of the T cell receptor (TCR)/antigen/human leukocyte antigen (HLA) complex revealed evidence that AAA encompasses a specific antigen-driven T cell response (4, 5). Studies discovered the clonal expansion of T cells in AAA lesions, linked AAA to specific HLA Class I and Class II types, and identified self- or non-self-antigens that may be associated with AAA (4, 6, 23).

The role of B cells in AAA is controversially discussed. B cell-derived immunoglobulins (Ig), such as IgM and IgG, localize in AAA tissue, where they promote inflammation and tissue degradation (10, 24). B cell depletion can prevent AAA growth in experimental models. One study showed that B cell depletion with an anti-CD20 antibody suppressed AAA growth in the angiotensin-II- and the elastase perfusion AAA model (25). Another study showed that AAA was induced by periaortic application of CaCl_2_, and significantly smaller AAAs in B cell-deficient muMT mice compared to wild-type (WT) mice were observed (10). Injection of polyclonal IgG antibodies into muMT mice resulted in AAA size comparable to WT mice indicating that IgG alone is sufficient to promote AAA development (10). In contrast, Meher et al. (11) could not observe differences in experimental AAA formation between muMT mice and WT mice and showed that adoptive transfer of B2 cells suppressed AAA formation and decreased infiltration of mononuclear cells into aneurysmal tissue. However, there is evidence that an autoimmune process directed against self-antigens in the aortic wall may play a role in AAA pathogenesis. Zhou et al. (23) identified a natural IgG antibody against fibrinogen in aortic tissues of elastase-induced AAA that induced AAA formation by activating the complement lectin pathway. Other findings suggest that a collagen-associated 80-kDA protein from the aneurysm wall is a potential target of the autoimmune response in AAA disease (26). IgG antibodies, including autoantibodies, have been isolated from the aortic wall of patients with AAA, and eight of 10 of these AAA wall IgGs reacted with an 80-kDa protein from aortic microfibrillar extracts shown by Western blotting. This protein was found to be located in the adventitial connective tissue matrix confirmed by immunohistochemistry (26). Further investigation of the role of T cells, B cells, and Ig involved in AAA is essential to improve the understanding of AAA pathogenesis. Clonality and diversity analysis of the adaptive immune receptor repertoire (AIRR) provide insights into disease mechanisms. Such analysis may further define the immunological status of an individual, thus proving useful for disease diagnosis and risk stratification (27).

AIRR sequencing is increasingly used to investigate lymphocyte dynamics in pathological contexts such as autoimmune and sterile inflammatory diseases, cancer, and infections (28). TCR and B cell receptors (BCR) are highly diverse heterodimers that recognize an immense variety of antigens (29). The receptors consist of a combination of heavy and light chains in the case of BCRs and a combination of α/β or γ/δ chains in the case of TCRs. The majority of TCRs expressed in T cells consist of a combination of α and β chains. The receptors are formed by variable, diversity, and joining (VDJ) recombination, which is the rearrangement of the V-, D-, and J-gene segments. For TCR, α chains, and BCR light chains, only V- and J-genes are involved in the recombination. Additional diversity is achieved by adding or deleting random nucleotides at the junction sites between the gene segments and by the chain pairing. Somatic hypermutation results in greater BCR diversity. Each receptor chain contains three hypervariable loops termed complementarity determining regions (CDR) that are required for the interaction of the receptors with the antigen. CDR3 is commonly used as a region of interest to determine T and B cell clones due to its high diversity and essential role in antigen binding (29–32). A clone is a set of cells expressing the same immune receptor, which implies that the receptors consist of the same V-, D-, and J-genes and encode the identical CDR3 nucleotide sequence. The AIRR is the union of all TCRs and BCRs of one individual and can change greatly with the onset and progression of diseases (29). The TCR repertoire within one individual is estimated to comprise 10^7^ in humans and 10^6^ in mice (33), whereas the estimated size of the B cell repertoire is 10^18^ in humans and 10^13^ in mice (34, 35).

Previously, TCRs were analyzed in aortic aneurysms, and their function has been investigated in mice and humans, yet no study addressed B cell clonality in aortic aneurysms. Li et al. (36) found clonal expansion of regulatory T cells (Treg) in mouse aortae after elastase-induced AAA formation, and several studies showed the presence of clonally expanded TCRs in aneurysmal lesions of patients with AAA or ascending thoracic aortic aneurysms, supporting the notion that AAA may be promoted by specific antigen-driven T cells (5, 37–39).

Single-cell RNA (scRNA) sequencing of TCRs and BCRs is a powerful tool for investigating the AIRR involved in AAA pathology. In comparison to bulk RNA sequencing, which yields a mixture of different gene expression profiles from the material studied, scRNA sequencing offers several advantages (40). scRNA sequencing provides information on TCR chain pairing and higher resolution and is more suitable for investigating the TCR specificity for an antigen of interest (41). However, there are also some limitations of scRNA sequencing. These challenges include the isolation of living single cells out of tissues, the lower output of sequenced cells compared to bulk sequencing, and higher costs (41). In particular, for scRNA sequencing of human AAA, only a small number of cells of interest is available for analysis, as only small sections of AAA can be collected during surgery (30). In mouse models, the whole AAA can be used, but the total amount of T and B cells is small for scRNA TCR and BCR sequencing. Zhao et al. (42) obtained approximately 3,000 cells, encompassing all present cell types, from topical elastase-induced AAA of 10 mice. Due to the small number of cells, biological interpretation of the sequenced TCR and BCR repertoire in AAA is challenging. In addition, standardized and uniform sample preparation, preprocessing of data, and bioinformatics workflow for data analysis are important to obtain robust and comparable data. There are already several guides (43), tools (44), and pipelines (45) for data analysis. However, in this paper, we highlight the limitations of scRNA TCR and BCR sequencing specifically in experimental AAA and provide a strategy for performing these experiments and for subsequent data analysis using a dataset we generated. The objective of this study is to guide and encourage fellow researchers to generate and evaluate scRNA TCR and BCR sequencing data, thereby enabling them to draw more significant conclusions.

## Methods

### Mice

Male C57BL/6J mice at the age of 10–11 weeks that were purchased from Janvier Labs (Saint-Berthevin, France) were used for experiments. All animal experiments were performed according to Animal Research: Reporting of In Vivo Experiments (ARRIVE) II guidelines and approved by LANUV (North Rhine-Westphalia State Agency for Nature, Environment and Consumer Protection) in accordance with the European Convention for the Protection of Vertebrate Animals used for Experimental and other Scientific Purposes (license approval number: 81-02.04.2018.A408). The mice were housed under standard laboratory conditions with a 12 h light/dark cycle and had *ad libitum* access to drinking water and standard chow.

### PPE perfusion model

To induce AAA in the mice, the PPE perfusion model was used as previously described by Pyo et al. (46). Briefly, the mice received analgesics by injecting 0.1 mg/kg body weight (bw) buprenorphine subcutaneously prior to surgery. The mice were anesthetized with isoflurane (initial 3%, then 1.5%) and oxygenated air. After the absence of the toe reflex, laparotomy was performed, and the proximal and distal infrarenal aorta was isolated and temporarily ligated. The aorta was punctured, a catheter was inserted, and the infrarenal part was perfused with sterile isotonic saline containing type I PPE (2.5–3 U/ml #E1250 Sigma-Aldrich, Burlington, MA, USA) or 0.9% NaCl (sham surgery) under 120 mmHG for 5 min. Elastase concentrations ranged from 2.5 to 3 U/ml depending on the batch number, as different concentrations were necessary to trigger the same AAA incidence and size. The aortic puncture was sutured, the ligations were removed, and the abdomen was closed. Afterward, the mice received buprenorphine (0.1 mg/kg bw, subcutaneously) if required in the first eight hours. In addition, the mice received buprenorphine (0.01 mg/ml) via the drinking water for three days. The mice were monitored regularly until the end of the experiment.

### Ultrasound imaging

Ultrasound was used to measure the aortic diameter prior to surgery and the AAA progression weekly. The Vevo 3100 high-resolution *in vivo* imaging system with a 25–55 MHz transducer (MX550D) (VisualSonics Inc., FUJIFILM, Toronto, Canada) was used for imaging. The mice were anesthetized with isoflurane and placed on a heated pad at 37°C. Aspiration rate, electrocardiogram, and body temperature were monitored during the entire time of imaging. To assess the aortic diameter, longitudinal B-mode images of the infrarenal aorta were acquired. The aortic diameter was analyzed from leading to leading edge (LTL) in three cardiac cycles at end-diastole using the Vevo LAB 5.6.0 software.

### Organ harvesting

The infrarenal aortae were harvested on days 3 (*n* = 5), 7 (*n* = 5), 14 (*n* = 2), and 28 (*n* = 4) after PPE surgery and on days 3 (*n* = 3) and 28 (*n* = 3) after sham surgery. In addition, the infrarenal aortae from non-treated C57BL/6J mice (*n* = 3) were pooled for two control samples. In total, we obtained 24 samples for scRNA sequencing. Approximately 10 min prior to organ harvesting, the mice were injected i.v. with 100 µl CD45-FITC antibody (#553079, BioLegend, dilution 1:1,000) to label circulating leukocytes. The mice were anesthetized and received analgesia with ketamine (100 mg/kg bw) and xylazine (10 mg/kg bw). After the absence of the toe reflex, blood was collected from the heart with a heparinized syringe. The thorax and abdomen were opened, the vena cava was cut, and the cardiovascular system was perfused with cold PBS through the left ventricle of the heart. The infrarenal part of the aorta was isolated by carefully removing all fatty tissue, collected, and stored in PBS on ice until further processing.

### Digestion of aortic tissue into single cells

The isolated infrarenal aortae were digested into single cells based on the protocol from Hu et al. (47). Briefly, the aortae were cut and transferred into an enzyme mix containing 500 U/ml collagenase I (Sigma-Aldrich, #C0130-100MG), 120 U/ml collagenase XI (Sigma-Aldrich, #C7657-25MG), 60 U/ml hyaluronidase I-S (Sigma-Aldrich, #H3506-100MG), and 60 U/ml Dnase I (Sigma-Aldrich, #11285932001) in Dulbecco's phosphate buffered saline (DPBS) containing calcium and magnesium supplemented with 20 mM HEPES (Thermo Fisher Scientific, #15630106). The aortae were incubated in the enzyme mix for 50 min on a shaker (600 rpm) at 37°C. The cell suspension was filtered through a 100 µm cell strainer (pluriSelect Life Science, Leipzig). The remaining aortic tissue was mashed with a syringe plunger through the cell strainer, which was rinsed several times with DPBS. After centrifugation (10 min, 450 × *g*, 4°C), the cells were resuspended in cold PBS and transferred into a 96-well plate.

For additional flow cytometric analysis, the cells were subsequently resuspended in RPMI 1,640 (Sigma-Aldrich) supplemented with 10% fetal calf serum (Sigma-Aldrich) and incubated on a shaker (600 rpm, 12 min, 37°C). Finally, cells were centrifuged (10 min, 450 × *g*, 4°C), resuspended in PBS, and transferred into a 96-well plate.

### Staining of single cells

The 96-well plate was centrifuged for 5 min at 500 × *g* and 4°C. The cells were stained with a staining mix containing Fc receptor blocker (TruStain FcX™, BioLegend, Amsterdam, Netherlands, 1:100), viability stain (Zombie Aqua™ and Zombie Green™ Fixable Viability Kit, BioLegend, 1:500), CD45-APC/cyanine 7 (BioLegend, clone 30-F11, dilution 1:200), TER-119-FITC (BioLegend, clone TER-119, 1:200), and C0443 CD41 (BioLegend, Barcode Sequence ACTTGGATGGACACT, 1:1,400) in PBS. In addition, an individual TotalSeq Hashtag antibody (BioLegend, TotalSeq™-C) was added to the single-cell suspension of each mouse. The hashtag antibodies allow the combination of samples from several mice in the same 10X sequencing run and are needed to demultiplex cells from individual mice. The cells from 10 mice were hashtagged with an antibody from the TotalSeq™-C series (BioLegend), respectively. Two additional hashtag antibodies were built by combining the antibodies MHC I-biotin (BioLegend, clone 28-8-6) and CD45-biotin (BioLegend, clone 30-F11) with the streptavidin-conjugated barcodes TotalSeq™ C971 or C972. The samples were stained with the staining mix and hashtag antibodies for 15 min at room temperature (RT) in the dark. After centrifugation (5 min, 500 × *g*, 4°C), the supernatant was discarded, and the cells were resuspended in MACS buffer (Miltenyi Biotec, #130-091-221) for following cell sorting.

For flow cytometric analysis, CountBright™ Absolute Counting Beads (Thermo Fisher Scientific) were added to every sample prior to staining to determine cell counts. The cells were centrifuged for 5 min at 500 × *g* and 4°C and stained with Fc receptor blocker (TruStain FcX^™^, BioLegend, 1:100) and viability stain (Zombie Aqua™ Fixable Viability Kit, BioLegend, 1:500) at RT for 10 min in the dark. After centrifugation (5 min, 500 × *g*, 4°C), the cells were stained with the following conjugated antibodies for 20 min at RT in the dark: CD3-APC (BioLegend, clone REA613, 1:200), CD11b-APC-Cy7 (BioLegend, clone M1/70, 1:200), CD19-FITC (BioLegend, clone MB19-1, 1:100), CD45-V450 (BioLegend, clone 30-F11, 1:200), Ly6C-PE (BioLegend, clone HK1.4, 1:133), Ly6G-PerCP-Cy5 (BioLegend, clone 1A8, 1:100), and NK1.1-PE-Cy7 (BioLegend, clone PK136, 1:200). The cells were centrifuged (5 min, 500 × *g*, 4°C) and resuspended in PBS with 0.5% bovine serum albumin. The samples were acquired with the BD FACSVerse™ Cell Analyzer (BD, Heidelberg, Germany), and data were analyzed with the FlowJo software v10.5.3.

### Cell sorting

Cell sorting was performed on a MoFlo XDP (Beckman Coulter, Krefeld, Germany). For every sample, up to 3,000 living CD45^+^ cells were sorted. If the samples did not reach the appropriate cell count, this was compensated by sorting more cells of other samples. The cells from all samples were combined into one reaction tube and centrifuged for 5 min at 500 × *g* at RT. The supernatant was removed, and the cells were resuspended in the MACS buffer. Recounting of the cells confirmed approximately 60,000 living cells.

### Generation of single-cell library

Single-cell libraries were generated with the 10X Chromium Controller system utilizing the Chromium Next GEM Single Cell 5′ Kit v2 (10X Genomics, Pleasanton, CA, USA) according to the instructions of the manufacturer. Sequencing was carried out on a NextSeq 550 system (Illumina Inc. San Diego, CA, USA) with a mean sequencing depth of ∼50,000 reads/cell for gene expression. The T cell and B cell libraries and the hashtag libraries were sequenced at ∼5,000 reads/cells.

### Processing of 10X genomics single-cell data

Raw sequencing data was processed using the 10X Genomics CellRanger software (v6.0.2). Raw BCL files were demultiplexed and processed to Fastq files using the CellRanger *mkfastq* pipeline. Alignment of reads to the mm10 genome and the corresponding VDJ gene references and UMI counting was performed via the CellRanger *multi* pipeline to generate a gene–barcode matrix.

### scRNA sequencing data analysis

R package *Seurat v4.0* (48) was used for analysis. First, the hashtag library was added as an assay to the metadata of the RNA library. The cells with less than 200 RNA counts and more than 30% mitochondrial RNA were excluded. Data were normalized and scaled with the functions *NormalizeData()* with the factor of 10.000, and *ScaleData()* was performed with all genes. Principal component analysis (75 dimensions), variable gene finding, cell clustering, and Uniform Manifold Approximation and Projection (UMAP) dimensional reduction (30 dimensions) were performed. Doublets were removed with *DoubletFinder v2.0* (49) and cells with positivity for more than one hashtag. T cells were defined by expression of *Cd3e*, *Cd3d*, *Cd3g*, and *Cd28*. B cells were defined by the expression of *Cd19*, *Cd79a*, and *Cd79b*. T and B cells were isolated bioinformatically and merged with the preprocessed scRNA TCR and BCR sequencing data.

### scRNA TCR and BCR sequencing analysis

Preprocessing of the scRNA TCR and BCR sequencing data included quality control and adding the library of the hashtag antibodies. Only receptors with exactly one alpha/heavy chain and one beta/light chain were used for analysis. Sequences of single chains, more than two chains per receptor, and non-matching chains were excluded from the analysis. The hashtag information was merged with the 10× output file to enable the assignment of TCRs and BCRs to the different mice. The *Immunarch package v 0.6.9* (50) was used to analyze CDR3 length distribution, clone abundance, repertoire overlap, germline gene V-gene usage, clonal expansion, and diversity estimation. We defined a clone as a set of T or B cells expressing the same receptor that consists of the same V-, D-, and J-genes and encodes an identical CDR3 nucleotide sequence.

### Statistics

Data are presented as absolute numbers or mean ± SD. Two-sample permutation-based Kolmogorov–Smirnov test was used to compare CDR3 length distributions with the function ks_test from R package “twosamples”. Bonferroni correction was used for multiple comparisons. To compare the correlation strength of the V-gene usage in the TCR alpha chain with the V-gene usage in the TCR beta chain, we compared the individual correlation coefficients with a two-tailed Mann–Whitney *U* test. For comparison of our AAA data with public databases, one-sided Fisher's exact test with Bonferroni correction was performed using the R v4.0 software. A detailed description of the database comparison can be found in the Supplementary Material. The results with *p* < 0.05 were considered significant.

## Results

### scRNA sequencing workflow

**Figure 1:**
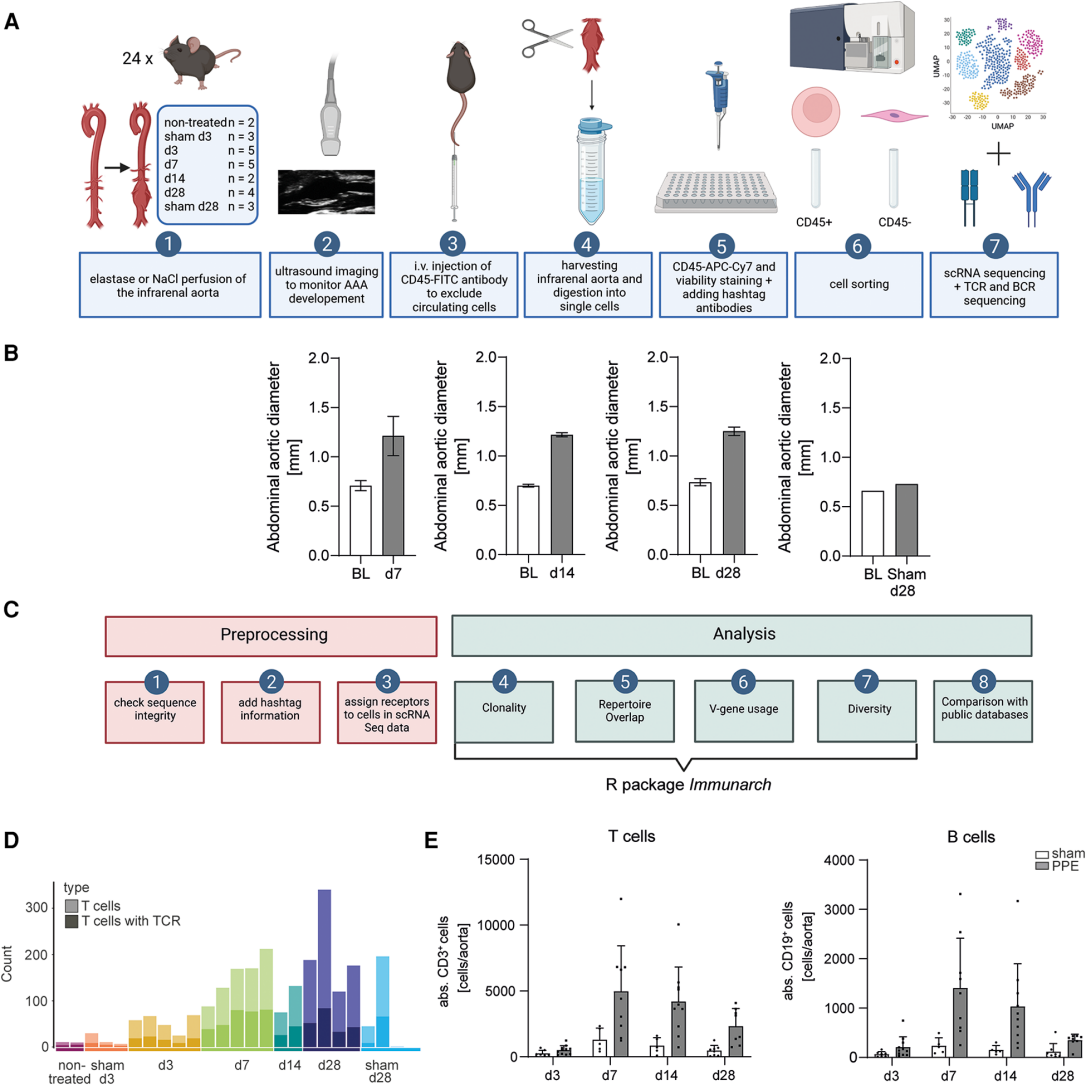
Experimental and bioinformatic workflow and quality control of the data. (**A**) Experimental workflow. An AAA was induced in mice via perfusion of the infrarenal aorta with elastase. NaCl perfusion of the aorta served as a sham-operated control. AAA development was monitored by ultrasound imaging. Prior to organ harvesting, the mice were intravenously injected with a fluorophore-labeled CD45 antibody. The harvested infrarenal aortae were digested into single cells and stained with antibodies and hashtags. The cells were sorted for living leukocytes and living non-leukocytes. scRNA sequencing and TCR and BCR scRNA sequencing were performed. (**B**) The abdominal aortic diameter was analyzed using ultrasound images of baseline (BL) and days 7 (*n* = 5), 14 (*n* = 2), and 28 (*n* = 2) post-PPE surgery and BL and day 28 (*n* = 1) post-sham surgery. (**C**) Bioinformatic workflow including preprocessing and data analysis. Three preprocessing steps were performed as quality control for the data. Only fully intact sequences were retained for analysis. The information for the hashtag antibodies was added to the TCR and BCR scRNA sequencing data. The receptors were assigned to the corresponding cells in the scRNA sequencing dataset. The main data analysis including clonality, repertoire overlap, V-gene usage, and diversity was performed with the immunarch R package. In addition, the data were compared with the public databases. (**D**) T cell amounts (light bar color) and T cell amounts exhibit an intact TCR (dark bar color) in aortic tissue on different AAA disease stages received from scRNA sequencing data. The amount of T cells increases with AAA progression. (**E**) Flow cytometric analysis of T and B cell amounts of aortic tissue 3 (*n* = 7), 7 (*n* = 5), 14 (*n* = 5), and 28 (*n* = 8) days after sham operation and 3 (*n* = 11), 7 (*n* = 9), 14 (*n* = 10), and 28 (*n* = 8) days after PPE-induced AAA formation.

The experimental workflow started with the induction of AAA by perfusing the infrarenal aorta of male C57BL/6J mice with elastase or NaCl (Figure 1A). AAA formation was monitored and analyzed via ultrasound imaging prior to surgery, at days 7, 14, and 28. The infrarenal aortae were harvested on days 3, 7, 14, and 28 after PPE surgery and on days 3 and 28 after sham surgery, and images were taken for macroscopic analysis. In addition, the infrarenal aortae from six non-treated C57BL/6J mice were harvested for sequencing, of which three were pooled into one sample. In total, 24 samples were subjected to scRNA sequencing and immunoreceptor analysis. All mice that underwent PPE surgery developed an AAA confirmed by ultrasound or macroscopic analysis of the infrarenal aorta (Figure 1B, Supplementary Figure S1). The aortae were harvested and digested into single-cell suspensions. Single cells were stained and sorted and then subjected to scRNA sequencing as well as TCR and BCR scRNA sequencing.

Consistent preprocessing of immune receptor scRNA sequencing data is crucial for comparable data. We suggest using only those immune receptor data, with all fragments intact and both chains (α/β for TCR or heavy/light for BCR) present. Receptors with only one, or more than two chains, that can appear in the data frame due to sequencing errors, were excluded. After that, the information of the hashtag antibodies was added to the data to assign an immune receptor specifically to one cell of a specific mouse. Subsequently, the receptors were assigned to the corresponding cell in our scRNA sequencing dataset. The majority of the immune receptor analysis such as clonality, repertoire overlap, V-gene usage, and diversity was performed with the R package *immunarch* (50). In addition, a comparison of the AAA data with public databases to identify disease-associated receptors was performed (Figure 1C).

### Analysis of scRNA TCR and BCR sequencing data

To ensure adequate data quality, we processed the TCR and BCR sequencing data and evaluated basic statistics. The raw data contained 3,370 TCR sequences [1,484 TCR alpha chains (TRA), 1,886 TCR beta chains (TRB)] and 1,745 BCR sequences [570 Ig heavy chains (IgH), 1,131 Ig kappa (IgK) light chains, 44 Ig lambda (IgL) light chains]. We observed a high number of immune receptors with only one sequenced chain and some receptors with more than two chains, which were excluded from subsequent analysis (Supplementary Figure S2). Only T cells with both productive TRA and TRB chains and B cells with both productive heavy and light chains were used for analysis. After that step, 2,296 TCR chains (1,148 pairs of TRA and TRB chains) and 980 BCR chains (490 pairs of heavy and light chains) remained. We next filtered for receptors that could be associated with a hashtagged cell and retained 2012 TCR chains and 770 BCR chains. Assignment of the immune receptors to the corresponding cells in our scRNA sequencing data revealed that only 47% of BCRs were expressed in B cells (defined by mRNA expression of *Cd19*, *Cd79a*, *Cd79b*), whereas the majority of TCRs (79%) was expressed in T cells (defined by mRNA expression of *Cd3e*, *Cd3d*, *Cd3g*, *Cd28*), and the remaining immune receptors were found on other cell types (Supplementary Figure S3). TCRs and BCRs not expressed in the respective lineage were excluded to avoid analysis of false positive receptors due to sequencing artifacts. The final analysis included 1,592 TCR chains (796 pairs of TCRs) and 358 BCR chains (179 pairs of BCRs).

We next compared the number of immune receptors with that of T and B cells, which were present in our scRNA sequencing dataset and displayed the distribution across the time points and samples (Figure 1D, Supplementary Figure S4 and Supplementary Table S2). Overall, there were fewer B cells (325) than T cells (2,376) in AAA tissue, and three out of 24 samples did not contain B cells (non-treated, d3, sham d28) (Supplementary Figure S4 and Supplementary Table S2). The total number of T and B cells increased with AAA progression until day 7 (Figure 1D, Supplementary Figure S4). We corroborated our findings by flow cytometry revealing a peak of lymphocytes at day 7 in AAA. Of note, only a few cells were detected in sham-operated mice (Figure 1E). A fully productive TCR could be assigned to 33.5% of the present T cells (2,376 T cells, 796 TCRs) (Figure 1D). Thus, a large proportion of TCR sequences present in AAA were missing due to inefficient sequencing. In our data, 55.1% of B cells had a matching BCR (325 B cells, 179 BCR). In four out of 24 samples, no BCRs could be detected, and these were sham-operated or early time points (non-treated, sham d3, d3, sham d28) (Supplementary Figure S4B and Supplementary Table S2).

### Estimating clonal expansion by spectratyping

**Figure 2:**
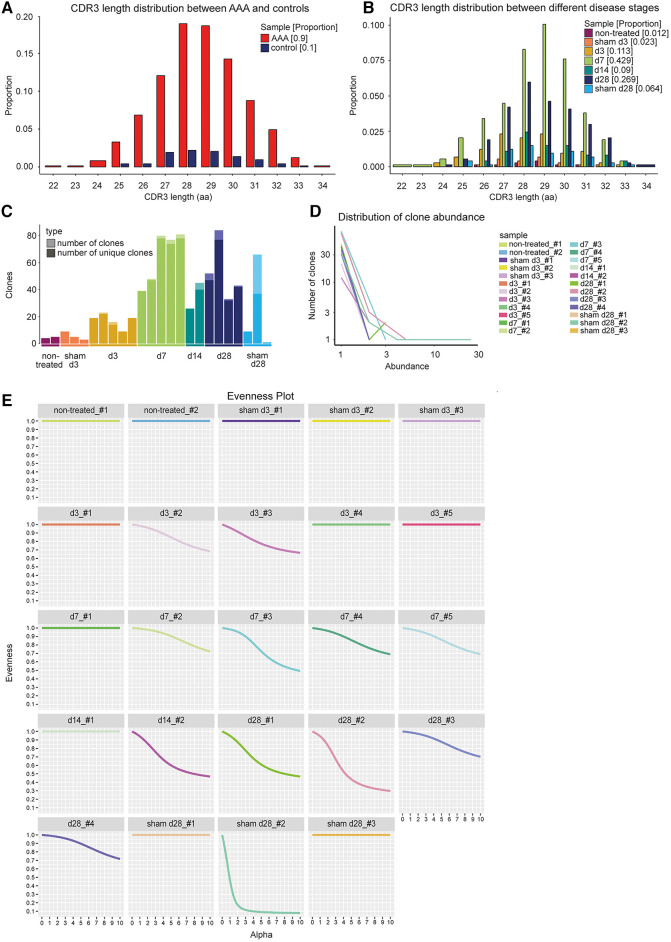
CDR3 length distribution and clone abundance indicate expanded T cell clones in elastase-induced aneurysm in mice. (**A**) No changes in CDR3 length distribution of TCRs (paired chains) between AAA including all different disease stages (red) and control samples including sham-operated and non-treated mice (blue). The proportion is plotted against the amino acid (aa) CDR3 length. (**B**) No alterations in CDR3 length distribution of TCRs (paired chains) between samples of different disease stages (days 3, 7, 14, 28) and sham-operated and non-treated samples (different colors). The proportion is plotted against the amino acid (aa) CDR3 length. (**C**) Amount of all TCR clones per sample (light bar color) including the amount of unique clones (dark bar color). The majority of TCR clones were found to be unique. Multiple copies of one clone appear only in one of the sham_d28 samples and in 11 of the AAA samples. (**D**) Line plot indicating the number and abundance of clones per sample. In the sham_d28 sample exhibiting clones with multiple copies, one clone is present 25 times. In contrast, in AAA samples, one clone only appears 2–5 times (**E**) Evenness plots indicating the extent of clonal expansion for every sample. One sham-operated sample 28 days after perfusion exhibits the highest clonality. The other control samples show no receptor clonality. Eleven AAA samples show some clonal expansion.

Spectratyping identifies the pattern of the CDR3 length distribution (51). Comparing the shape of the CDR3 length distribution between control and disease can indicate the presence of clonal expansion in a repertoire. Deviations from the normal pattern might be due to the high frequency of a specific CDR3 sequence and are therefore associated with clonal expansion (31). We compared the CDR3 length distribution of TCRs in AAA across all time points and controls using the two-sample permutation-based Kolmogorov–Smirnov test (Figure 2A). The resulting *p*-value of 0.92 suggests that the CDR3 length followed the same distribution in AAA and controls. To compare the CDR3 length distribution between the different disease stages, we performed pairwise two-sample permutation-based Kolmogorov–Smirnov tests and used Bonferroni correction for multiple comparisons. We did not observe significant differences between the CDR3 length distribution at the different time points (Figure 2B).

Receptor clonality can also be investigated by determining the number of unique clones and the clone abundance. A clone was defined as a set of cells expressing the same receptor that consists of the same V-, D-, and J-genes and encodes an identical CDR3 nucleotide sequence. The majority of TCRs in AAA, sham-operated, and non-treated aortae were unique (Figure 2C, Supplementary Table S3). Only one sample of the sham-operated and non-treated aortae and 11 AAA samples contained T cell clones (Figure 2C, Supplementary Table S3). However, the clones were infrequent in AAA samples (occurring 2–5 times), whereas one of the four T cell clones present encompassed 25 cells in one sham-operated sample (Figure 2D, Supplementary Table S3). The frequencies of the T cell clones, their V-, D-, and J-genes of alpha and beta chain, and their CDR3 nucleotide and amino acid sequences are shown in Supplementary Table S4. The extent of receptor clonality can be indicated with an evenness profile of the repertoire (27). The alpha values represent different diversity indices with different weights on expanded clones. Higher alpha values give more weight to expanded clones, while alpha = 0 weights every clone equally regardless of its frequency. Therefore, high receptor clonality is indicated as a highly uneven curve, and no receptor clonality is associated with a completely even curve (Figure 2E). In our study, the sham-operated d28 sample, in which one clone was identified 25 times (Figure 2D), exhibited also the highest clonality, whereas all other sham-operated or non-treated samples showed no clonality (Figure 2E). In addition, 11 AAA samples from different time points showed a lower extent of receptor clonality.

### Investigating the TCR repertoire similarity

**Figure 3:**
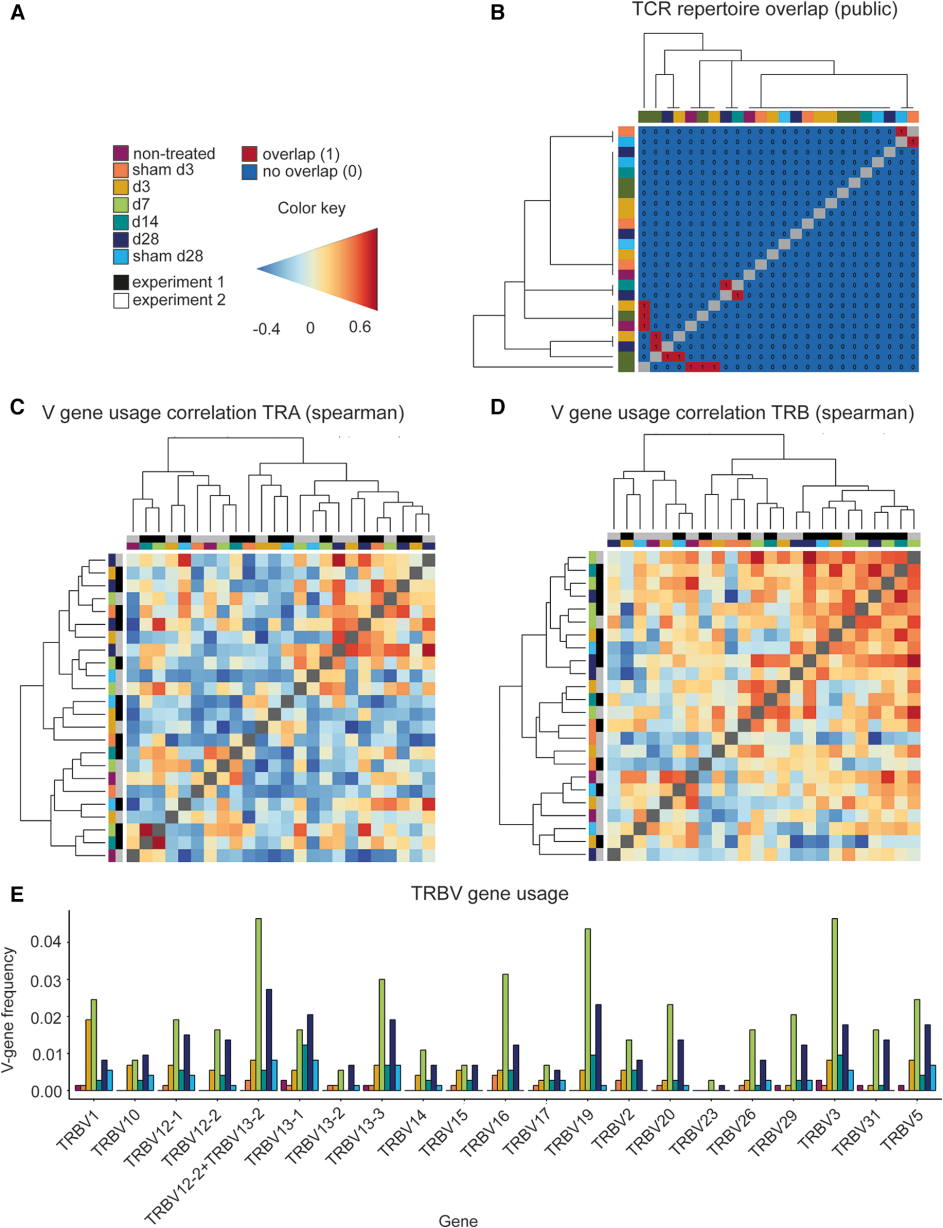
Comparing the different TCR repertoires reveals shared TCR sequences, a high correlation of the V-gene usage and several frequently used TRBV genes in AAA. (**A**) Legend and color code. (**B**) Analysis of the TCR repertoire overlap shows that in 7 instances two AAA samples contain one equal TCR. The color code in the heatmap indicates the different samples. The V-gene usage of the TCR alpha chain (**C**) and of the TCR beta chain (**D**) is correlated and hierarchical clustered between the different samples using spearman correlations. Color gradient indicates the level of correlation (blue = negative correlation, red = positive correlation). The color code on the axes indicates the different samples. (**E**) Distribution and frequency of TRBV genes occurring in all samples. Frequently used TRBV genes in AAA samples are TRBV3, TRBV19, and TRBV12-2+TRBV13-2 at day 7, 14 and 28. TRA, TCR alpha chain; TRAV, TCR alpha chain v gene; TRB, TCR beta chain; TRBV, TCR beta chain v gene.

Repertoire overlap analysis is commonly used to identify “public” TCRs that are shared between individuals (52). The R package *immunarch* provides several methods to measure receptor similarities between individuals. Using the function “public” specified, the exact number of shared immune receptors between different repertoires, thereby revealing that in seven instances, a TCR sequence was shared by two AAA samples (Figure 3A,B). In addition, repertoire similarity can be investigated by identifying TCRs of different individuals containing the same V region genes. Fragments of V region genes are classified into families according to their nucleotide sequence similarity (at least ∼70%). Specific V-gene usage patterns have been associated with different diseases and were shown to change in response to therapeutic approaches (53, 54). In our data, the V-gene usage of the beta chain (TRBV) correlated stronger than the V-gene usage of the alpha chain (TRAV) of the TCR (two-tailed Mann–Whitney *U* test, *p* = <0.0001, Figures 3C,D). A deeper analysis of the distribution and frequency of used TRBV genes revealed a high usage of TRBV3, TRBV19, and TRBV12-2 + TRBV13-2 in AAA samples at days 7, 14, and 28 (Figure 3E). TRBV12-2 + TRBV13-2 is a term for a common alternate splicing between the first exon of TRBV12-2 and the second exon of TRBV13-2. TRBV19 was present in 5 and TRBV3 in 2 of the expanded clones. Further Vbeta genes that were used by 2–3 of the expanded TCRs are TRBV10, TRBV13-1, TRBV13-5, TRBV2, TRBV20, and TRBV29 (Supplementary Table S4).

### Dataset comparison with public TCR and BCR databases

**Figure 4:**
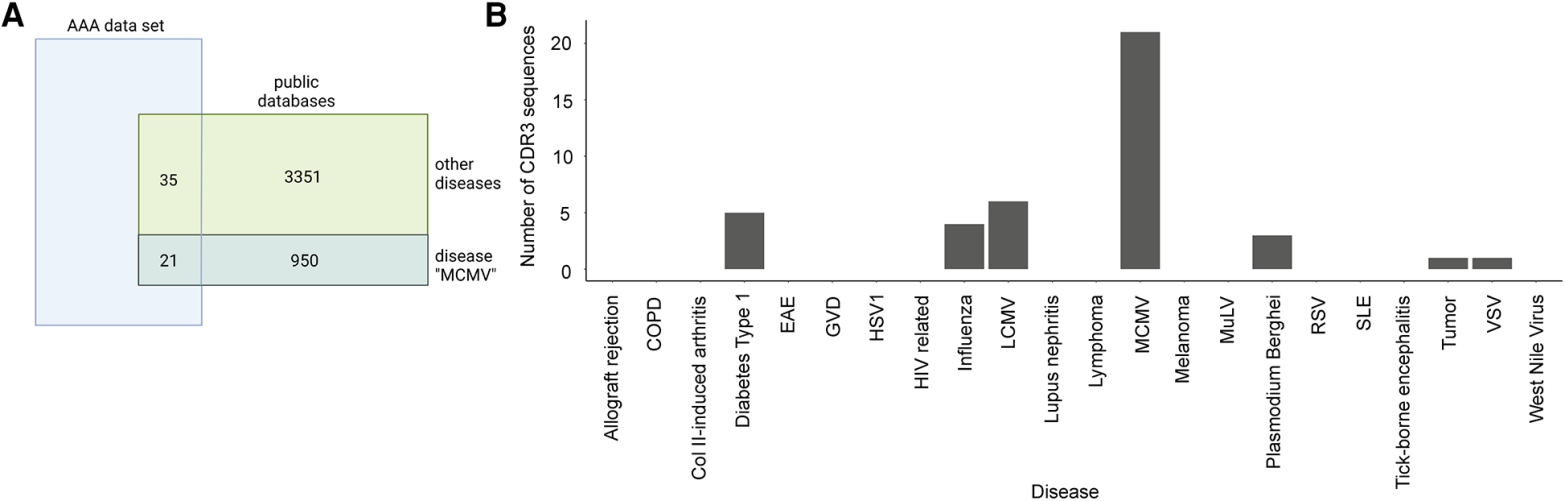
Overlap of AAA-associated CDR3 sequences with public databases. (**A**) Venn diagram indicating the overlap of our TCR data with the public TCR databases (VDJdb and McPAS-TCR) representative for the disease MCMV. Our data shares 21 CDR3 sequences with the public databases that are associated with MCMV and 35 CDR3 sequences that are associated with other diseases. One-sided Fisher's exact test with Bonferroni correction for multiple comparisons was used to examine the overrepresentation of TCR clones in our dataset that are associated with diseases or antigens. (**B**) Barplot displaying the number of sequences per disease in our data. COPD, chronic obstructive pulmonary disease; Col II, collagen II; EAE, experimental autoimmune encephalomyelitis; GVD, graft vs. host disease; HSV1, herpes simplex virus 1; HIV, human immunodeficiency virus; LCMV, lymphocytic choriomeningitis virus; MCMV, murine cytomegalovirus; MuLV, murine leukemia virus; RSV, respiratory syncytial virus; SLE, systemic lupus erythematosus; VSV, vesicular stomatitis virus.

We compared the presence of CDR3 sequences in our dataset with the two public TCR databases VDJdb (55) and McPAS-TCR (56) to investigate if TCR clones in our dataset are associated with other diseases or antigens (57). VDJdb is a curated database of TCR sequences with known antigen specificities containing TCR information of three different species (*Homo sapiens*, *Macaca mulatta*, and *Mus musculus*) and various diseases (55). McPAS-TCR is a database of TCR sequences found in T cells that were associated with various pathological conditions in humans and mice (56). TCRs with less than four amino acids and diseases with less than five TCRs were excluded. Accordingly, 5,206 TCRs were found in influenza (3,156 TCRs), lymphocytic choriomeningitis virus (LCMV) (151 TCRs), murine cytomegalovirus (MCMV) (1,463 TCRs), *Plasmodium berghei* (245 TCRs), respiratory syncytial virus (RSV) (125 TCRs), and vesicular stomatitis virus (VSV) (66 TCRs) for VDJdb, and 3,530 TCRs that were assigned to 21 different diseases/pathogens were used for analysis for McPAS-TCR (Supplementary Table S5). After merging, the two databases and filtering for unique CDR3 sequences, we obtained 4,331 CDR3 sequences for comparison with the AAA dataset. One-sided Fisher's exact test was used to examine the overrepresentation of TCR clones in our dataset that are associated with diseases or antigens according to the two databases (Figure 4A). Our dataset shared 55 CDR3 sequences with the public databases, which were assigned to MCMV, LCMV, influenza, *Plasmodium berghei*, VSV, diabetes type 1, and tumor. The obtained *p*-values were adjusted for multiple testing using Bonferroni correction. Bonferroni correction resulted in no significant *p*-values indicating there were no TCR clones overrepresented in our dataset that are associated with diseases or antigens according to the two databases (Figure 4B, Supplementary Table S6).

### No clonal expansion or repertoire overlap of BCRs in elastase-induced aneurysm in mice

**Figure 5:**
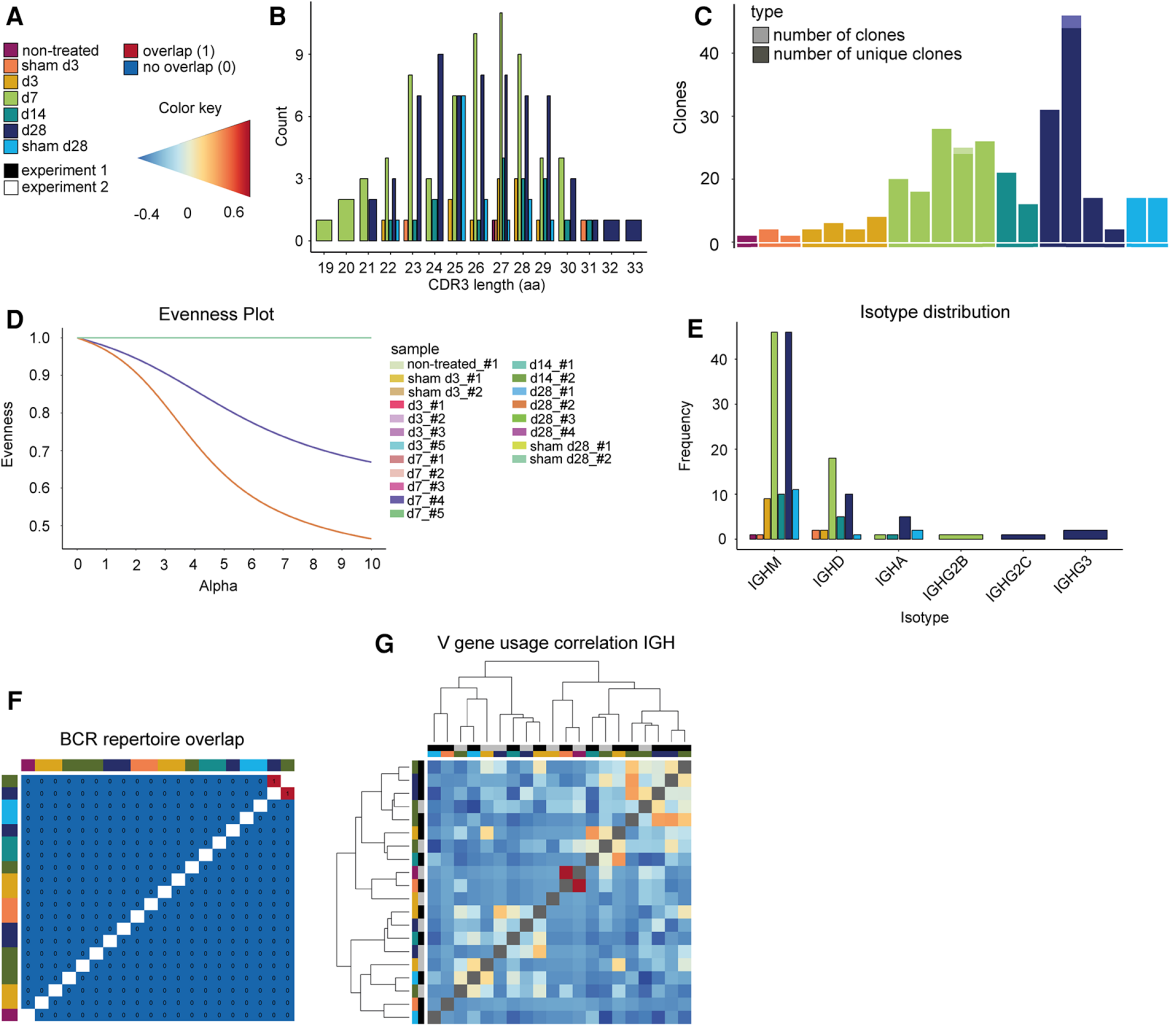
Elastase-induced AAA shows no BCR clonality. (**A**) Legend and color code. (**B**) Histogram indicating the CDR3 length distribution of the BCRs (paired chains). The proportion is plotted against the amino acid (aa) CDR3 length. The color indicates whether the TCRs belong to an aorta isolated at days 3, 7, 14, or 28 after PPE or sham surgery or to a non-treated aorta. (**C**) Barplot displaying the number of all BCR clones (pale color) and only unique clones (vibrant color) per mouse. Almost all BCR clones are unique. (**D**) Evenness plot indicating the extent of clonal expansion for every sample. (**E**) Barplot indicating the isotype distribution of the BCR heavy chain (IGH) in the different conditions. The most frequent isotypes for all conditions are IGM and IGD. (**F**) BCR repertoire overlap is displayed in a heatmap. The color bars on the top and left side of the heatmap indicate the time point. (**G**) Heatmap presenting the Spearman correlation and hierarchical clustering of the BCR heavy chain (IGH) V-gene usage of the different samples. The color gradient indicates the level of correlation (blue = weak correlation, red = strong correlation). The color bars on the top and left side of the heatmap indicate the experiment and the time point.

The CDR3 length distribution of the BCRs showed no differences between AAA and control at the disease stages (Figure 5A,B, pairwise two-sample permutation-based Kolmogorov–Smirnov test resulted in no significant differences). Furthermore, 98% of BCR clones were unique (176 of 179 clones were unique). As mentioned before, BCRs were present in only 20 of 24 samples in our dataset. The samples that lacked BCRs were control samples or early disease stages that are known to contain few B cells overall (non-treated, sham d3, sham d28, d3). BCRs that appear more than once were found in two of these 20 samples. One day 28 sample contained one BCR that was present thrice, and one day 7 sample had one BCR that appeared twice (Figure 5C). The evenness profile likewise indicated a higher clonality for these two AAA samples in comparison to all other samples that showed no clonality (Figure 5D, Supplementary Figure S5). Next, we investigated the isotype distribution of the BCR heavy chains. The most frequent isotype was IGHM, followed by IGHD (Figure 5E). The similarity measurement of the BCR repertoire present in the different samples showed that two AAA samples (days 7 and 28) share one BCR (Figure 5F). Otherwise, there was no similarity between the different samples. The correlation of V-gene usage between the different samples was likewise low (Figure 5G). The strongest, yet still weak correlation, was found between one non-treated control and one d3 sample (*r* = 0.701). Overall, these data revealed no evidence for clonality among B cells in AAA.

## Discussion

We assessed TCR and BCR clonality in elastase-induced AAA in mice at different disease stages using scRNA TCR and scRNA BCR sequencing. Our results show no differences in CDR3 length distribution of TCRs and BCRs between the different disease stages, indicating no strong clonal expansion of immune cell receptors in elastase-induced AAA. The clone abundance analysis likewise revealed no clonal expansion of BCRs in AAA. We found expanded T cell clones in 68% of AAA samples and no clonality in control samples except for one. A comparison of the immune receptor repertoires showed a low similarity between the individual samples. Spearman correlation to compare the V-gene usage between the different AAA samples and controls revealed that the V-gene usage of the TCR beta chain correlates stronger than the V-gene usage of the TCR alpha chain. The most frequently used V-genes in the TCR beta chain in AAA are TRBV3, TRBV19, and TRBV12-2 + TRBV13-2. A comparison of TCR clones identified by us revealed no overrepresentation of TCR clones associated with diseases or antigens annotated in two public databases. The main Ig isotype in our BCR dataset is IgM followed by IgD. Although this may prompt speculation of enrichment of B1 cells that predominantly express IgM, the overall scRNA-sequencing dataset suggests that B2 cells are approximately 20-fold more frequent than B1 cells. Notably, this corroborates previous reports of B2 dominating the B cell pool in mouse AAA (11).

### Clonal expansion, public TCRs, and convergent T cells

Antigen recognition by immune cell receptors activates naive lymphocytes prompting them to proliferate. This process is termed clonal expansion and enables a targeted, adaptive immune response. However, the term clonal expansion remains strongly debated as there is no clear and consensus definition. Lu et al. investigated T cell clonality in aneurysmal lesions of AAA patients and defined clonal expansion as the presence of multiple identical copies of TCR transcripts (37). They reasoned that the size of the T cell repertoire makes it unlikely that multiple identical copies of a TCR transcript would be found by chance in an independent sample of T cells (37). According to this definition, we found clonal expansion in 11 of 16 AAA samples and one of eight control samples.

Public TCRs are shared across different individuals due to VDJ recombination biases and might target common antigens (52). The repertoire similarity of the AAA samples was low. In seven instances, a TCR sequence and only one BCR sequence were shared by two individual AAA samples. Next to clonal expansion and public TCRs, there is also T cell convergence. Convergent T cells are cells expressing TCRs with identical CDR3 amino acid sequences and variable genes but different CDR3 nucleotide sequences (58). Convergent T cells arise due to codon degeneracy and can be observed in almost every individual. Pan and Li (58) showed that convergent T cells are different from public TCRs and seem to be antigen-specific. According to their results, TCR convergence might be a better indicator of antigen specificity than clonal expansion. Since convergent T cells constitute only a small proportion of the total population of T cells, studies of TCR convergence require a large number of sequenced T cells. We did not find convergent T cells in our dataset, probably due to the small number of T cells but expect that investigating T cell convergence in larger datasets may be a feasible and worthwhile approach to address this important component of an antigen-specific T cell response.

### Clonal expansion in human AAA and atherosclerosis

Studies with patients demonstrated the presence of clonally expanded TCRs in AAA or ascending thoracic aortic aneurysms (TAA), supporting the paradigm of AAA as a disease driven by an antigen-specific T cell response (5, 37–39). In particular, clonal expansion of TCR beta (5, 37) and alpha (38) chains was demonstrated in AAA lesions of patients while others reported clonal expansion of γ/δ T cells in AAA (5). Furthermore, TCRs were investigated in different types of TAA (patients with Marfan syndrome, familial TAA, and sporadic aneurysm), and the results indicate a similar clonal nature of the TCRs present in TAA (39). He et al. (39) found a preferential usage of the V-genes Vb22 and Vb25 in lesions from patients with TAA. Lu et al. (37) reported multiple appearances (at least twice) of TRBV3 in 60% of AAA patients. Atherosclerotic vascular disease, which is also a risk factor for AAA development while it also shares some common (immuno-) pathophysiological pathways (59), is likewise associated with T cell expansion and clonality. In particular, TCRs containing Vβ6 are expanded in atherosclerotic lesions of mice (60). Moreover, a decreased diversity of the TCR β chain repertoire was shown in human atherosclerotic plaques due to the expansion of a few T cell subclones (61).

### Limitations of the current study

The main limitation of the current study is the relatively small number of lymphocytes resulting in potential undersampling. The limited number of T and B cells resulted from the naturally scarce source (i.e., minimal aneurysm size in mice), from additional sorting procedures (sorting of all leukocytes and not specifically T and B cells), and not fully efficient sequencing. Indeed, we had to exclude many TCR and BCR sequences from the data due to inefficient sequencing. The undersampling leads to the issue that the TCR and BCR copies in our data do not represent the real absolute number of copies present in AAA and even the ratio of the various clones to each other does not reflect the real ratio (62). Accordingly, this data should be interpreted cautiously and presents restricted value for biological interpretation. Further experiments are needed to verify the evidence of T cell clonality in elastase-induced AAA. Until now, mouse models have been standard for studying mechanisms of human pathophysiology. However, there are considerable differences between species regarding genetics, physiology, and immunology, which have to be considered. Although the PPE model is the mouse model most closely resembling human AAA, it does not fully mimic the complexity of AAA development in humans (15, 63). Human AAA features a complex and long-lasting disease development that is only partially resembled in experimental rodent models that aim to recapitulate disease patterns in a few weeks. Thus, effects observed in mice have to be extrapolated with caution to human aneurysmal and atherosclerotic disease.

### Future perspectives and recommendations

To overcome the problem of undersampling, we suggest sorting at least 5,000–10,000 B and T cells instead of including all leukocytes. We detected by flow cytometry on average 120–430 B cells and 400–1,600 T cells per mg AAA tissue depending on the stage of AAA development, while lymphocyte numbers are lower in control conditions (e.g., native or -sham-operated mice; on average 120–300 B cells and 400–500 T cells/mg aortic tissue). Thus, pooling of aneurysms from several mice is necessary to obtain a sufficient number of lymphocytes. In this case, we recommend the use of Hashtag antibodies before pooling to enable the assignment of the lymphocytes to the corresponding mouse and to monitor clonality for each individual. Next, the choice of experimental model should be carefully considered, as each model has limitations and mimics specific features of human AAA (15). Li et al. (36) induced AAA in mice with elastase and CaPO_4_, performed scRNA sequencing combined with TCR sequencing of 41,341 CD4+ T cells isolated from AAA, and found a clonal expansion of Treg. This suggests that the number of analyzed cells is an important factor for investigating TCR clonality and a high number of cells facilitates the identification of clonal expansion. Higher cell counts increase the likelihood of detecting rare TCR and BCR clones and allow additional investigation of T cell convergence. We would like to recommend prioritized sequencing of the TCR and BCR libraries and to include DNA-barcoded antibodies against B cells (CD19) and T cells (CD3), which allows for superior identification of subpopulations in comparison to identification by mRNA expression of feature genes. In addition to analyzing the aneurysmatic tissue itself, future studies may also include sequencing of secondary lymphoid organs (e.g., draining lymph nodes) to identify changes in lymphocyte clonality, migration, and activity. scRNA TCR and BCR sequencing has the advantage of a high-throughput, multi-parametric analysis of target cells. Drop-sequencing approaches, such as the commercially available 10X Genomics solution used here, allow us to interrogate the transcriptome, TCR, and BCR of 1,000 cells simultaneously. However, there is a high dropout in detecting lowly expressed genes, which bears limitations: (1) this technology is particularly advantageous in describing a diverse cell population, while other approaches might be superior in studying transcriptional changes in related subpopulations, (2) full-length transcripts of TCRs and BCRs for both chains might not be detectable in all cells (we identified full-length TCRs and BCRs in 33.5% and 55.1% of all cells). To uncover detailed transcriptional changes in T cell subpopulations, this approach could be complemented by sorting these cells and performing bulk transcriptomics, which delivers a deeper insight. In addition, beta repertoire sequencing can be used to approximate T cell clonality on a global level and confirm observations made by scRNA TCR sequencing. However, this method does not provide information about the transcriptome of an individual cell or the corresponding paired TCR alpha chain, thus not reflecting the true complex clonality.

For the comparison of datasets from different research groups, it is important to have standardized workflows for sample preparation and the preprocessing of the data. Accordingly, we present an example and detailed workflow for the preprocessing steps. We recommend including only immune receptors with two productive chains that can be assigned to a B or T cell. In addition to clonal expansion and repertoire similarity, future analyses should also address T cell convergence.

## Conclusion

In conclusion, we found evidence of clonal expansion of T cells, but not of B cells, in experimental elastase-induced AAA. Due to the small number of cells further experiments are needed to verify the evidence of T cell clonality. Since other studies found TCR clonality in AAA lesions of patients and considering the paradigm of an autoimmune response in aneurysmal disease, further examination of TCR and BCR clonality is important. Our findings imply that a precise characterization of TCR and BCR distribution requires a more extensive number of lymphocytes to prevent undersampling and to allow for the detection of rare clones and convergent T cells. This paper provides an in-depth analysis of TCR and BCR sequencing data, emphasizes the potential drawbacks and constraints of these experiments, and offers recommendations for future investigations in this area.

## Data Availability

The datasets presented in this study can be found in online repositories. The names of the repository/repositories and accession number(s) can be found here: https://doi.org/10.5281/zenodo.7942455.
